# Repeated cross-sectional analysis of hydroxychloroquine deimplementation in the AHA COVID-19 CVD Registry

**DOI:** 10.1038/s41598-021-94203-7

**Published:** 2021-07-23

**Authors:** Steven M. Bradley, Sophia Emmons-Bell, R. Kannan Mutharasan, Fatima Rodriguez, Divya Gupta, Gregory Roth, Ty J. Gluckman, Rashmee U. Shah, Tracy Y. Wang, Rohan Khera, Pamela N. Peterson, Sandeep Das

**Affiliations:** 1 Healthcare Delivery Innovation Center, Minneapolis Heart Institute, 920 East 28th Street, Suite 300, Minneapolis, MN 55407 USA; 2 Institute for Health Metrics and Evaluation, University of Washington, Seattle, USA; 3 Northwestern University Feinberg School of Medicine, Chicago, IL USA; 4 Division of Cardiovascular Medicine, Cardiovascular Institute, Stanford University, Stanford, CA USA; 5 Department of Medicine, Emory Heart and Vascular Center, Emory University School of Medicine, Atlanta, USA; 6 Center for Cardiovascular Analytics, Research and Data Science (CARDS), Providence Heart Institute, Portland, OR USA; 7 Division of Cardiovascular Medicine, University of Utah School of Medicine, Salt Lake City, UT USA; 8 Duke Clinical Research Institute, Duke University, Durham, NC USA; 9 Section of Cardiovascular Medicine, Department of Internal Medicine, Yale School of Medicine, New Haven, CT USA; 10 Center for Outcomes Research and Evaluation, Yale-New Haven Health, New Haven, CT USA; 11 Denver Health Medical Center, Denver, CO USA; 12 University of Colorado Anschutz Medical Center, Aurora, CO USA; 13 Center for Innovation and Value at Parkland, University of Texas Southwestern Medical Center, Dallas, TX USA

**Keywords:** Viral infection, Psychology and behaviour

## Abstract

There is little data describing trends in the use of hydroxychloroquine for COVID-19 following publication of randomized trials that failed to demonstrate a benefit of this therapy. We identified 13,957 patients admitted for active COVID-19 at 85 U.S. hospitals participating in a national registry between March 1 and August 31, 2020. The overall proportion of patients receiving hydroxychloroquine peaked at 55.2% in March and April and decreased to 4.8% in May and June and 0.8% in July and August. At the hospital-level, median use was 59.4% in March and April (IQR 48.5–71.5%, range 0–100%) and decreased to 0.3% (IQR 0–5.4%, range 0–100%) by May and June and 0% (IQR 0–1.3%, range 0–36.4%) by July and August. The rate and hospital-level uniformity in deimplementation of this ineffective therapy for COVID-19 reflects a rapid response to evolving clinical information and further study may offer strategies to inform deimplementation of ineffective clinical care.

## Introduction

In the care of patients with COVID-19, anecdotal reports and in vitro data were made available in March of 2020 that suggested the potential for hydroxychloroquine to reduce disease severity^1,2^. Subsequent observational and randomized studies of hydroxycholorquine for COVID-19 have failed to demonstrate benefit, with the first peer-reviewed randomized trial published on May 14, 2020^3,4^. The threshold at which evidence is considered sufficient to merit practice change may vary between hospitals, particularly in light of statements by high-ranking public officials in support of hydroxychloroquine for COVID-19^5^. Furthermore, once practice patterns are established, they can be difficult to change^6^. We hypothesized that hospital rates of hydroxchloroquine use would vary over time with the potential for these findings to inform future studies of deimplementation strategies for ineffective care.

## Methods

### Study design, setting, and participants

The American Heart Association (AHA) COVID-19 Cardiovascular Disease Registry captures data on all patients hospitalized for active COVID at participating hospitals, including patient demographics, comorbidities and risk factors, hospital treatments, and clinical outcomes. Additional details on the registry have been published^7,8^. As we sought to describe trends in hospital-level use of hydroxycholorquine, we restricted our analysis to hospitals with at least 10 cases of COVID-19 submitted to the registry between March 1 and August 31, 2020, to avoid inflating variation as a function of small sample size. Among patients with multiple admissions in the registry, we restricted our analysis to the first admission. We excluded patients with preexisting lupus, rheumatoid arthritis, or use of hydroxychloroquine prior to admission where it may reflect baseline therapy.

### Statistical analysis

We describe the proportion of hospitalized patients who received hydroxychloroquine during the study period overall and by two-month calendar intervals. These intervals were selected to maintain sample size at the facility level for comparison of hydroxychloroquine use. We describe the median, interquartile range, and overall range of hydroxychloroquine use by hospital for the overall study period and by two-month calendar intervals.

## Results

**Figure 1:**
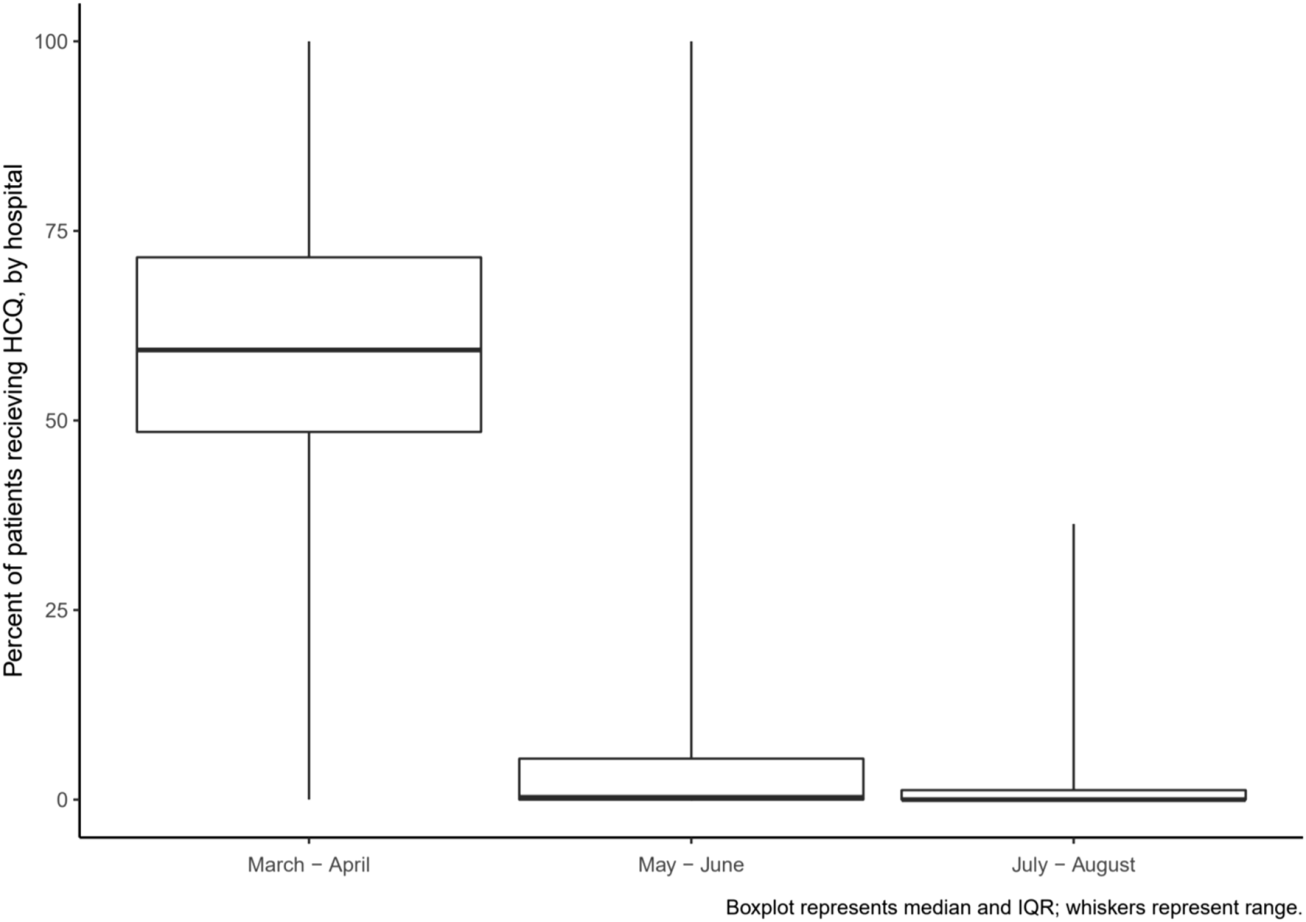
Hospital-level hydroxychloroquine use over time.

We identified 13,957 patients admitted for COVID-19 in the U.S. between March 1 and August 31, 2020, at 85 hospitals in 60 counties and 28 states. Patient characteristics by hydroxycholorquine are shown in Table 1^8^. The overall proportion of patients receiving hydroxychloroquine was 37.6%, with a peak of 55.2% in March and April and decreasing to 4.8% in May and June and 0.8% in July and August. At the hospital-level, the median use of hydroxychloroquine for the period of study was 35.6% (IQR 14.2–37.7%, range 0–95.5%). In March and April, the hospital-level median use was 59.4% (IQR 48.5–71.5%, range 0–100%) and decreased to 0.3% (IQR 0–5.4%, range 0–100%) by May and June and 0% (IQR 0–1.3%, range 0–36.4%) by July and August (Fig. 1).

**Table 1:** Patient characteristics and outcomes by hydroxychloroquine use.

	HCQ administered (n = 5167)	HCQ not administered (n = 8569)
**Demographics**		
Age, mean (SD)	61.9 (16.3)	61 (18.7)
Female, n (%)	2139 (41.4%)	4053 (47.3%)
**Admission features**		
BMI, mean (SD)	30.8 (8.1)	30.4 (8.4)
SpO2, mean (SD)	92.8 (7.1)	94.4 (6.3)
Heart rate, mean (SD)	96.2 (19.2)	93.3 (19.9)
Systolic BP, mean (SD)	131.3 (23.2)	130.9 (24.4)
Creatinine, mean (SD)	1.8 (7.1)	1.8 (4.4)
Pulmonary infiltrates, n (%)	4215 (81.6%)	5290 (61.7%)
Supplemental O2, n (%)	1233 (23.9%)	2350 (27.4%)
**Past medical history, n (%)**		
CABG or PCI	332 (6.4%)	560 (6.5%)
Cancer	536 (10.4%)	1027 (12%)
Cerebrovascular disease	362 (7%)	1069 (12.5%)
Chronic kidney disease	594 (11.5%)	1166 (13.6%)
Diabetes	1887 (36.5%)	3113 (36.3%)
Heart failure	442 (8.6%)	1087 (12.7%)
Hypertension	3086 (59.7%)	5032 (58.7%)
Pulmonary disease	942 (18.2%)	1503 (17.5%)
Smoking	320 (6.2%)	607 (7.1%)
**Hospital outcome**		
Placed on ventilator, n (%)	1513 (29.3%)	1254 (14.6%)
Inpatient death, n (%)	1047 (20.3%)	1164 (13.6%)

*BMI* body mass index, *BP* blood pressure, *CABG* coronary artery bypass grafting, *HCQ* hydroxychloroquine, *n* number, *PCI* percutaneous coronary intervention, *O*_*2*_ oxygen, *SpO*_*2*_ oxygen saturation.

## Discussion

In a large national study of patients hospitalized for COVID-19, we found use of hydroxychloroquine was common in March and April of 2020, but varied at the hospital-level. Use of hydroxychloroquine dropped precipitously after April and hospital-level variation decreased. The rate and hospital-level uniformity in deimplementation of this ineffective therapy for COVID-19 reflects a rapid response to evolving clinical information^9,10^.

Early in the COVID-19 pandemic, use of hydroxychloroquine was supported on the basis of in vitro data, small case series, approval of this therapy by governmental agencies, and statements from public leaders. In this early phase of the pandemic, the median hospital rate of hydroxychloroquine use was 59% with rates of use varying by facility. These findings mirror prior studies of the early phase of the pandemic^11,12^. Prior studies of uptake of new medical therapies and devices have demonstrated similar variation in rates of uptake with use influenced by the strength of scientific evidence, exposure to marketing of the new therapy, and support of the new device or therapy by national programs^13,14^. The observed variation in hydroxycholoroquine use early in the pandemic may reflect the lack of formal national programs related to the therapy in the U.S. and the lack of high quality data in the form of randomized controlled trials demonstrating benefit. Our study lacks data on the presence or absence of local clinical champions for the therapy and provider perception of statements by public leaders related to hydroxycholorquine use that may have also contributed to the observed variation. Similarly, the high degree of interest by non-medical personnel and patients may have also contributed to provider knowledge and attitudes in the use of hydroxychloroquine.

Following the publication of randomized trials that failed to demonstrate benefit for hydroxychloroquine in the care of COVID^3,4^, we observed a precipitous and uniform drop in the use of this therapy. Typically, as new evidence emerges that casts doubt on existing treatments, change to reduce or eliminate use of ineffective therapy is often slow and inconsistent and requires external support, active engagement with providers, and the development of training and education to impact a provider’s knowledge and attitude toward practice behaviors^6,15–17^. This slow pace of change in the use of ineffective and unnecessary care puts patients at risk and contributes to high costs of healthcare^6^. As a result, unnecessary care remains prevalent and a focus of efforts to improve healthcare value^9,15^.

The change in the use of hydroxychloroquine for COVID-19 is atypical in both the rapidity and uniformity of discontinuation of an ineffective practice. This may in part reflect that use of hydroxychloroquine was not an ingrained practice behavior such that typical processes needed to support deimplementation were not required^6^. Similarly, it may reflect that provider’s knowledge and attitudes on hydroxycholorquine were malleable^17^, given the evolving understanding of the pandemic and the high level of interest in COVID-19 from both medical and non-medical personnel. Finally, the lack of U.S. national programs related to the use of hydroxychloroquine may have facilitated a rapid transition, as change was not dependent on the dissolution of a national program. As health systems continue to look for strategies to reduce and eliminate ineffective care delivery, further study of adoption and deimplemenation of therapies during the pandemic may offer additional insights^10^.
